# Differential utilization patterns of dissolved organic phosphorus compounds by heterotrophic bacteria in two mountain lakes

**DOI:** 10.1093/femsec/fiw139

**Published:** 2016-06-15

**Authors:** Carina Rofner, Ruben Sommaruga, María Teresa Pérez

**Affiliations:** Institute of Ecology, Lake and Glacier Ecology Research Group, University of Innsbruck, Technikerstr. 25, 6020 Innsbruck, Austria

**Keywords:** ATP, glucose-6-phosphate, glycerol-3-phosphate, freshwater bacteria, AcI *Actinobacteria*, R-BT *Betaproteobacteria*

## Abstract

Although phosphorus limitation is common in freshwaters and bacteria are known to use dissolved organic phosphorus (DOP), little is known about how efficiently DOP compounds are taken up by individual bacterial taxa. Here, we assessed bacterial uptake of three model DOP substrates in two mountain lakes and examined whether DOP uptake followed concentration-dependent patterns. We determined bulk uptake rates by the bacterioplankton and examined bacterial taxon-specific substrate uptake patterns using microautoradiography combined with catalyzed reporter deposition–fluorescence *in situ* hybridization. Our results show that in the oligotrophic alpine lake, bacteria took up ATP, glucose-6-phosphate and glycerol-3-phosphate to similar extents (mean 29.7 ± 4.3% *Bacteria*), whereas in the subalpine mesotrophic lake, ca. 40% of bacteria took up glucose-6-phosphate, but only ∼20% took up ATP or glycerol-3-phosphate. In both lakes, the R-BT cluster of *Betaproteobacteria* (lineage of genus *Limnohabitans*) was over-represented in glucose-6-phosphate and glycerol-3-phosphate uptake, whereas AcI *Actinobacteria* were under-represented in the uptake of those substrates. *Alphaproteobacteria* and *Bacteroidetes* contributed to DOP uptake proportionally to their *in situ* abundance. Our results demonstrate that R-BT *Betaproteobacteria* are the most active bacteria in DOP acquisition, whereas the abundant AcI *Actinobacteria* may either lack high affinity DOP uptake systems or have reduced phosphorus requirements.

## Abstract (precis)

In phosphorus-limited mountain lakes, the most abundant taxa, the AcI lineage of *Actinobacteria* and the R-BT cluster of *Betaproteobacteria*, exhibit strikingly different dissolved organic phosphorus uptake patterns.

## INTRODUCTION

In oligotrophic freshwater ecosystems, phosphorus (P) is often the element limiting primary and heterotrophic bacterial production (Carlson 1977; Vadstein 2000). Although inorganic phosphorus (P_i_) is considered to be the preferred P source for microbes (Björkman and Karl 1994; Karl 2000), dissolved organic phosphorus (DOP) often represents most of the dissolved phosphorus pool in aquatic ecosystems (Minear 1972; Karl *et al.*^2001^). Among the large diversity of DOP compounds, ATP is known to be readily available for the majority of planktonic microorganisms (Holm-Hansen and Booth 1966; Berman 1988; Bentzen, Taylor and Millard 1992). In these studies, bacteria outcompeted phytoplankton in ATP acquisition at low ambient concentrations, and thus it was assumed that they might harbor high affinity transport systems to sequester DOP. However, few studies have considered other DOP substrates, although compounds such as glycerol phosphate and glucose phosphate are known to reduce the P_i_ demand of microbial communities (Argast and Boos 1980; Berman 1988; Cotner and Wetzel 1992).

Bacteria can adapt rapidly to P_i_-deficient conditions because P_i_ sensors and response proteins act as transcriptional activators of the Pho regulon that codes for several genes involved in the cleavage and incorporation of DOP compounds (Wanner 1993; Luo *et al.*^2009^). Two mechanisms are commonly used for DOP incorporation. For instance, phosphoesters such as ATP are cleaved by extracellular hydrolases (e.g. alkaline phosphatases) and the released P_i_ residues are subsequently taken up into the cell (Jansson, Olsson and Pettersson 1988; Ammerman and Azam 1991). However, other DOP compounds enter the bacterial cell intact via specific transport systems (Brzoska *et al.*^1994^; Yang, Wang and Metcalf 2009) and are either hydrolyzed intracellularly or directed to metabolic pathways (Wanner 1993). Bacteria living under P limitation can express genes responsible for different DOP uptake mechanisms (Luo *et al.*^2009^; Vila-Costa *et al.*^2012^). However, there is an uneven distribution of P-related functional genes among bacteria, which suggests that distinct bacterial groups might rely on specific DOP compounds or rather, that they differ in their ability to utilize DOP (Luo *et al.*^2009^; Vila-Costa *et al.*^2012^).

Here, we hypothesized that P-limited lake bacteria respond rapidly to the addition of different DOP compounds and determined whether this contribution changes with increasing substrate concentrations. Further, we tested whether individual bacterial groups differ in their ability to utilize these compounds as they potentially exhibit different DOP uptake characteristics. We determined the bulk uptake rates of ATP, glucose-6-phosphate (Glu6P) and glycerol-3-phosphate (Gly3P), offered at three different concentrations, by the bacterial community of a mesotrophic subalpine and an oligotrophic alpine lake. Additionally, we examined the uptake of these three substrates by individual bacterial groups using microautoradiography (MAR) combined with fluorescence *in situ* hybridization with catalyzed reporter deposition (CARD-FISH).

## EXPERIMENTAL PROCEDURES

### Study sites and sample collection

Uptake experiments were conducted in two mountain lakes located in the Austrian Alps, namely the oligotrophic alpine lake Gossenköllesee (GKS, 47°13′N, 11°01′E) located at 2417 m a.s.l., and the mesotrophic subalpine lake Piburgersee (PIB, 47°11′N, 10°53′E) located at 913 m a.s.l. GKS is a holomictic dimictic lake with a maximum depth of 9.9 m and a lake area of 1.7 ha. The lake is ice-covered for up to 7 months. PIB is a meromictic dimictic lake with a maximum depth of 24.6 m and an area of 13.4 ha. The ice-cover in PIB typically lasts from early December until April. Further information on lake characteristics and seasonality can be found elsewhere (Tolotti and Thies 2002; Sommaruga and Augustin 2006).

Due to the very large sample number to process and to count for MAR-CARD-FISH (2 lakes × 2 depths × 3 substrates × 3 concentrations × 3 replicates × 6 16S rRNA probes = 648 samples), both lakes were sampled only once during the stratified period (mid-August in GKS and mid-October in PIB). At that time, water temperature was similar between lakes, making enzymatic and bacterial activity more comparable. At each sampling date, water samples from the epilimnion (1 m) and the aerobic hypolimnion (8 m in GKS, 15 m in PIB) were collected from the central area of the lakes using a 5 L Schindler–Patalas sampler. Water samples (1 L) for bulk uptake experiments and MAR-CARD-FISH incubations, as well as for dissolved organic carbon (DOC) were collected in pre-combusted (450°C, 4 h) borosilicate glass bottles. Samples to determine total phosphorus (TP) and total dissolved phosphorus (TDP) concentrations were collected in 1 L polyethylene bottles pre-rinsed with 1 M HCl. Subsamples for DOC and TP/TDP analyses were processed as previously described (Hörtnagl, Pérez and Sommaruga 2010).

### Incubations for microautoradiography

To assess DOP utilization patterns by individual bacterial groups, the following radiochemicals were used for microautoradiography (MAR) (specific activity 20 Ci mmol^−1^; American Radiolabeled Chemicals): [^3^H]adenosine triphosphate (ATP), [^3^H]glucose-6-phosphate (Glu6P) and [^3^H]glycerol-3-phosphate (Gly3P). Due to the impossibility of purchasing all three substrates with ^32/33^P-label, we used instead ^3^H-labeled substrates to assure comparability of uptake patterns. For every substrate, three different concentrations were used (0.2, 1 and 5 nM) to check whether their uptake follows a concentration-dependent pattern, as DOP *in situ* concentrations are known to fluctuate (e.g. year-round bioavailability of ATP; Rofner, Sommaruga and Pérez 2016). All MAR incubations were run in triplicate (20 ml for GKS, 10 ml for PIB) plus a control sample that was killed 15 min before radiotracer inoculation (2% formaldehyde). Samples were incubated at *in situ* temperature in the dark for 45 min (ATP) or 60 min (Glu6P and Gly3P) and incubations were stopped by adding formaldehyde (2% final concentration). Samples were fixed overnight at 4°C and two subsamples (10 ml for GKS, 5 ml for PIB) were filtered the next day onto 0.22 μm polycarbonate white filters (Millipore GTTP) followed by subsequent rinsing with 5–10 ml of 0.22 μm filtered MQ-water. Filters were stored frozen (–20°C) until further processing.

### Bulk uptake rates

The bulk uptake rates of [^3^H]ATP, [^3^H]Glu6P and [^3^H]Gly3P were assessed by measuring the radioactivity retained onto 0.22 polycarbonate white filters (Poretics). Duplicate samples (10 ml for GKS, 5 ml for PIB) plus one formaldehyde-killed control were incubated with the radiolabeled substrates as described in the previous section. Filters were dissolved in 5 ml scintillation cocktail (Ready-Safe, Beckman Coulter) and their radioactivity assessed after 15 h on a Beckman LS 6000IC scintillation counter.

### MAR-CARD-FISH procedure

CARD-FISH was done as described in Pernthaler, Pernthaler and Amann (2002) using the modified permeabilization protocol of Sekar *et al.* (2003). The most common bacterial groups/clades in the study lakes were targeted by the following horseradish peroxidase-labeled rRNA probes (ThermoHybaid): EUB I–III for the domain *Bacteria* (Daims *et al.*^1999^), ALF968 for *Alphaproteobacteria* (Neef 1997), BET42a for *Betaproteobacteria* (Manz *et al.*^1992^) and its R-BT cluster with R-BT065 (lineage of the genus *Limnohabitans*) (Šimek *et al.*^2001^), CF319a for *Bacteroidetes* (Manz *et al.*^1996^), and AcI-852 for AcI *Actinobacteria* (Warnecke *et al.*^2005^). Filter hybridization and preparation of CARD-FISH slides was done as previously described (Pérez and Sommaruga 2011). In total, 216 individual filter sections were prepared for CARD-FISH and 648 for MAR-FISH.

Microautoradiogaphy was done according to Tabor and Neihof (1982) after transferring cells onto coverslips (Cottrell and Kirchman, 2000, 2003). The coverslips were dipped into a molten autoradiography emulsion (Kodak, type NTB), exposed for 10 d at 4°C and developed afterwards according to the manufacturer's instructions. Enumeration of slides was done according to Pérez and Sommaruga (2011) without subtraction of blank counts because previous results from our experiments have shown that <1% of 4′,6-diamidino-2-phenylindole (DAPI)-stained cells are labeled in the blank.

CARD-FISH filters were counterstained with DAPI (1 μg ml^−1^) and were also used to assess bacterial abundance. About 400–1300 homogeneously distributed cells per filter were counted manually by epifluorescence microscopy (Zeiss Axiophot 2).

### Statistical analyses

A one-way analysis of variance (ANOVA) or a *t*-test (two sample test) were run on PAST.exe (Ver. 2.17c) to detect significant differences among sample means of bacterial groups taking up the DOP substrates. Sample means were compared between bacterial groups, concentrations, depths, lakes and substrates. When significant differences (*P* < 0.05) were found, a *post hoc* test (Tukey) was applied. Normal distribution of data was visually checked with histograms, normal probability plots and the Shapiro–Wilk test. Data were log-transformed, when found to be not normally distributed. A linear regression analysis was done in PAST.exe to detect if the proportions of active cells within individual bacterial groups changed significantly with increasing substrate concentrations.

## RESULTS

### Study sites

Water temperature was slightly higher in August in GKS than in October in PIB (Table 1). In both lakes, pH values were close to neutral, but nutrient concentrations were generally higher in PIB than in GKS (Table 1). DOC concentrations were slightly higher in the epilimnion of the lakes, whereas TDP and TP concentrations were higher in the hypolimnion. Bacterial abundance was rather similar at both depths in PIB (mean ∼1.38 × 10^6^ cells ml^−1^), but it was two-fold higher in the hypolimnion of GKS than in the epilimnion (Table 1).

**Table 1.** Summary of the physicochemical and biological parameters measured in lakes Gossenköllesee (GKS) and Piburgersee (PIB). Temp: water temperature; TDP: total dissolved phosphorus; TP: total phosphorus; DOC: dissolved organic carbon; BA: bacterial abundance.

Lake	Date	Depth (m)	Temp (°C)	pH	TDP (nM)	TP (nM)	DOC (μM)	BA (cells ml^−1^)
GKS	16.08.2013	1	14.3	7.15	25.83	87.17	34.63	4.78 × 10^5^
		8	8.7	7.19	45.20	122.68	24.56	9.87 × 10^5^
PIB	18.10.2013	1	11.3	7.6	67.8	190.48	185.08	1.31 × 10^6^
		15	5.2	6.96	93.63	400.34	153.78	1.45 × 10^6^

### Lake-specific phylogenetic affiliation of heterotrophic bacteria and their contribution to dissolved organic phosphorus uptake

Probe EUBI-II-III detected ∼80% of DAPI-stained cells in both lakes (Table 2). The actinobacterial lineage AcI was the most abundant group, comprising about 40% of DAPI-stained cells in the epilimnion and ca. 35% of DAPI-stained cells in the hypolimnion of the lakes. *Betaproteobacteria* was the second most abundant group and showed similar percentages (∼29% of DAPI-stained cells) at both depths in GKS, but not in PIB, where it was more abundant in the hypolimnion. The R-BT cluster comprised about one-third of *Betaproteobacteria* in GKS and ca. one-fourth in the epilimnion of PIB; however, they represented a small fraction of *Betaproteobacteria* in the hypolimnion of the latter lake. *Alphaproteobacteria* showed a similar distribution in both lakes, representing 8–10% of DAPI counts in the epilimnion and 4–5 times less in the hypolimnion. *Bacteroidetes* represented on average 9.8% and 3.2% of DAPI counts in GKS and PIB, respectively, although in both lakes their relative abundance was higher in the epilimnion as compared with the hypolimnion.

**Table 2.** Structure of the bacterial community in lakes Gossenköllesee (GKS) and Piburgersee (PIB). EUBI-II-III (*Bacteria*), ALF968 (*Alphaproteobacteria*), BET42a (*Betaproteobacteria*), R-BT065 (R-BT cluster of *Betaproteobacteria*), CF319a (*Bacteroidetes*) and AcI-852 (AcI lineage of *Actinobacteria*). The mean (*n* = 9 replicates) relative abundance of probe-specific hybridized cells is given as the percentage of DAPI counts ±SD.

Lake	Date	Depth (m)	EUBI-II-III	ALF968	BET42a	R-BT065	CF319a	AcI-852
GKS	16.08.2013	1	86.89 ± 1.59	8.04 ± 0.18	29.08 ± 1.62	12.96 ± 2.19	11.03 ± 1.27	39.32 ± 1.14
		8	71.23 ± 3.91	1.46 ± 0.71	28.03 ± 1.11	10.07 ± 1.43	8.56 ± 0.82	32.54 ± 2.82
PIB	18.10.2013	1	79.05 ± 4.32	10.30 ± 2.18	21.21 ± 1.25	5.67 ± 1.34	4.04 ± 1.09	40.30 ± 2.86
		15	82.24 ± 4.87	2.24 ± 0.84	34.58 ± 1.68	5.15 ± 1.19	2.36 ± 0.20	36.83 ± 2.55

**Figure 1.**
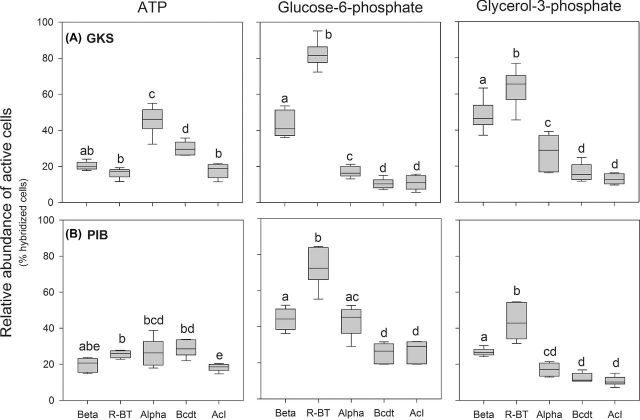
The relative abundance of cells within the bacterial groups (as % hybridized cells) taking up ATP, glucose-6-phosphate and glycerol-3-phosphate at three different concentrations and in both depths of GKS (**A**) and PIB (**B**). Boxplots show the 25–75 percentiles and the median. Different letters above the boxplots indicate significant differences in substrate uptake among the bacterial groups (Tukey's pairwise *post hoc* test; significance level of 0.05), whereas identical letters indicate similar substrate uptake patterns. *Betaproteobacteria* (Beta) and its R-BT cluster (R-BT), *Alphaproteobacteria* (Alpha), *Bacteroidetes* (Bcdt), and AcI *Actinobacteria* (AcI).

**Figure 2.**
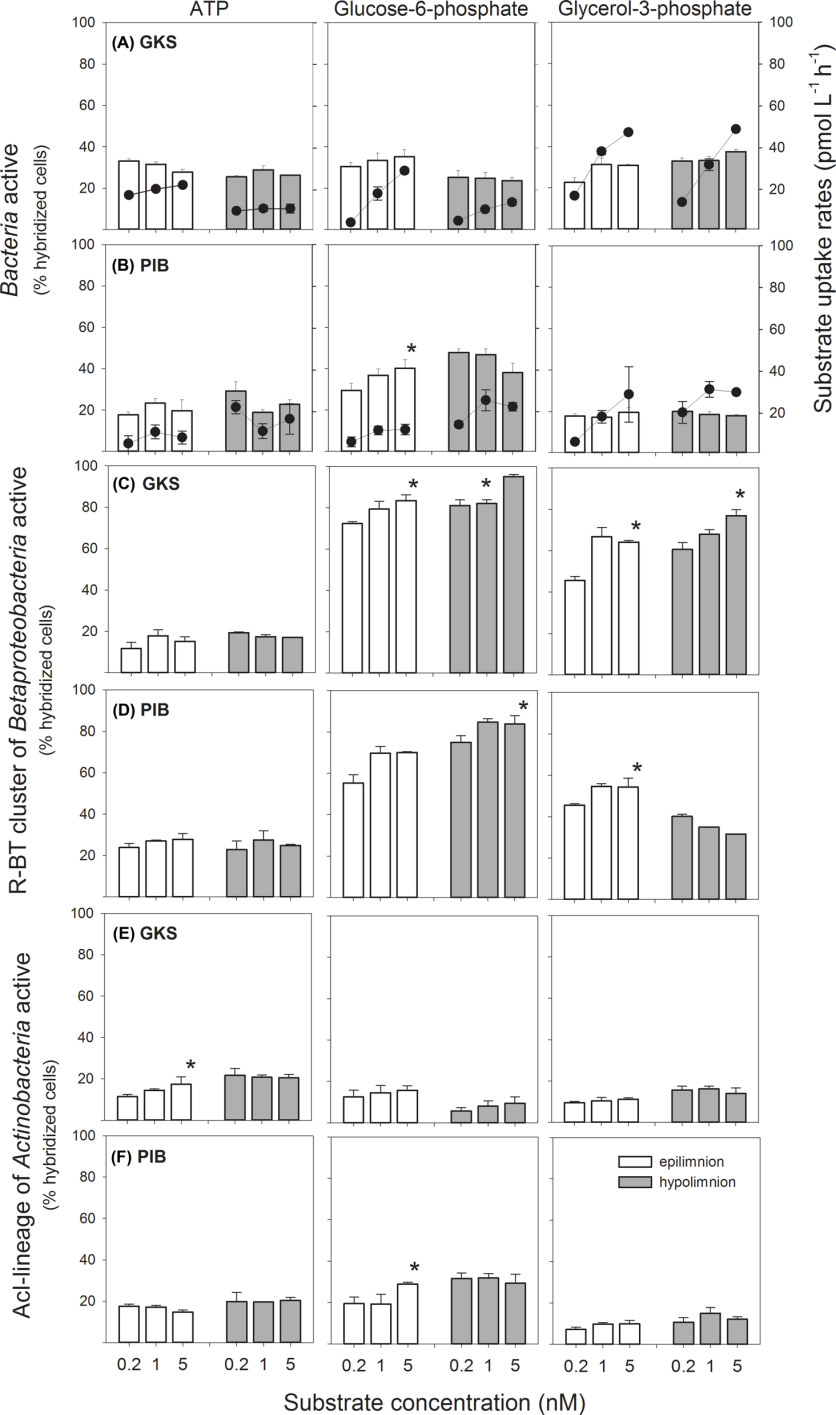
Bar charts representing the percentage of *Bacteria*, the R-BT cluster of *Betaproteobacteria* and the AcI lineage of *Actinobacteria* (as % hybridized cells) taking up ATP, glucose-6-phosphate and glycerol-3-phosphate at the three concentrations added (0.2, 1, 5 nM) in the epilimnion (open bars) and hypolimnion (filled bars) of GKS (**A**, **C**, **E**) and PIB (**B**, **D**, **F**). Values are the mean of triplicate incubations ±1 SD. Asterisks indicate significant changes in active cells due to increasing substrate concentrations. Data points above the bars represent the mean bulk substrate uptake rates determined from duplicate incubations ±1 SD.

The examined bacterial groups exhibited substrate-specific uptake patterns that were similar in both lakes (Fig. 1A and B). *Betaproteobacteria* and its R-BT cluster yielded the highest proportions of cells taking up Glu6P and Gly3P in the lakes, though more cells were labeled positive in GKS than in PIB. For both groups, the proportions of cells positive for ATP uptake represented ca. 20% of hybridized cells. *Bacteroidetes* and AcI *Actinobacteria* showed similar Glu6P and Gly3P uptake patterns (Fig. 1) and were often different from those of other bacterial groups (ANOVA, *P* < 0.01). In general, AcI *Actinobacteria* was weakly represented in the uptake of any substrate (range: ∼6–22% of hybridized cells) with the exception of Glu6P in PIB (range: ∼19–32% of hybridized cells; Figs 1B and 2F). By contrast, more *Bacteroidetes* cells were labeled positive for ATP uptake (22–36%) than for Gly3P or Glu6P uptake in the lakes. Around 30–55% of *Alphaproteobacteria* incorporated ATP and Gly3P (hypolimnion) in GKS and Glu6P in PIB.

### Dissolved organic phosphorus uptake in relation to substrate concentration

In GKS, the percentage of bacterial cells taking up the three DOP compounds was very similar (Fig. 2A; average: ATP 28.81 ± 3.03%, Glu6P 28.77 ± 4.94%, Gly3P 31.39 ± 4.87%), whereas in PIB ca. 40% of bacterial cells took up Glu6P and between 18 and 24% of *Bacteria* took up ATP and Gly3P (Fig. 2B). The Glu6P and Gly3P bulk uptake rates increased greatly at higher substrate concentrations in both lakes (Fig. 2A and B), whereas the relative abundance of bacterial cells taking up these substrates remained rather constant or increased significantly for Glu6P uptake in the epilimnion of PIB (Fig. 2B; *R*^2^ = 0.78; *P* < 0.05). By contrast, ATP bulk uptake rates and the proportions of cells positive for ATP uptake increased slightly or fluctuated without clear pattern with increasing substrate concentration.

The R-BT cluster of *Betaproteobacteria* was particularly active in Glu6P and Gly3P uptake and higher substrate concentrations yielded often significantly higher percentages of positive cells (Fig. 2C and D; *R*^2^ = 0.50–0.88, *P* < 0.05). This effect was more pronounced in GKS than in PIB. By contrast, higher ATP concentrations did not affect the R-BTs activity and the proportions of ATP-labeled cells remained low (range: GKS 12–19% hybridized cells; PIB 23–28% hybridized cells).

In general, the proportions of actinobacterial cells positive for a DOP compound either slightly increased at higher substrate concentrations or remained constant (Fig. 2E and F). The AcI lineage of *Actinobacteria* showed similar percentages of cells labeled positive for ATP and Gly3P in both lakes, whereas more cells were labeled positive for Glu6P in PIB than in GKS (range: PIB 19–32% hybridized cells; GKS 6–15% hybridized cells). In GKS, the proportions of actinobacterial cells labeled positive for ATP increased significantly in the epilimnion (*R*^2^ = 0.69, *P* < 0.05), whereas in the epilimnion of PIB this trend was significant for Glu6P uptake (*R*^2^ = 0.73, *P* < 0.05).

### Dissolved organic phosphorus uptake in relation to group-specific abundance

**Figure 3.**
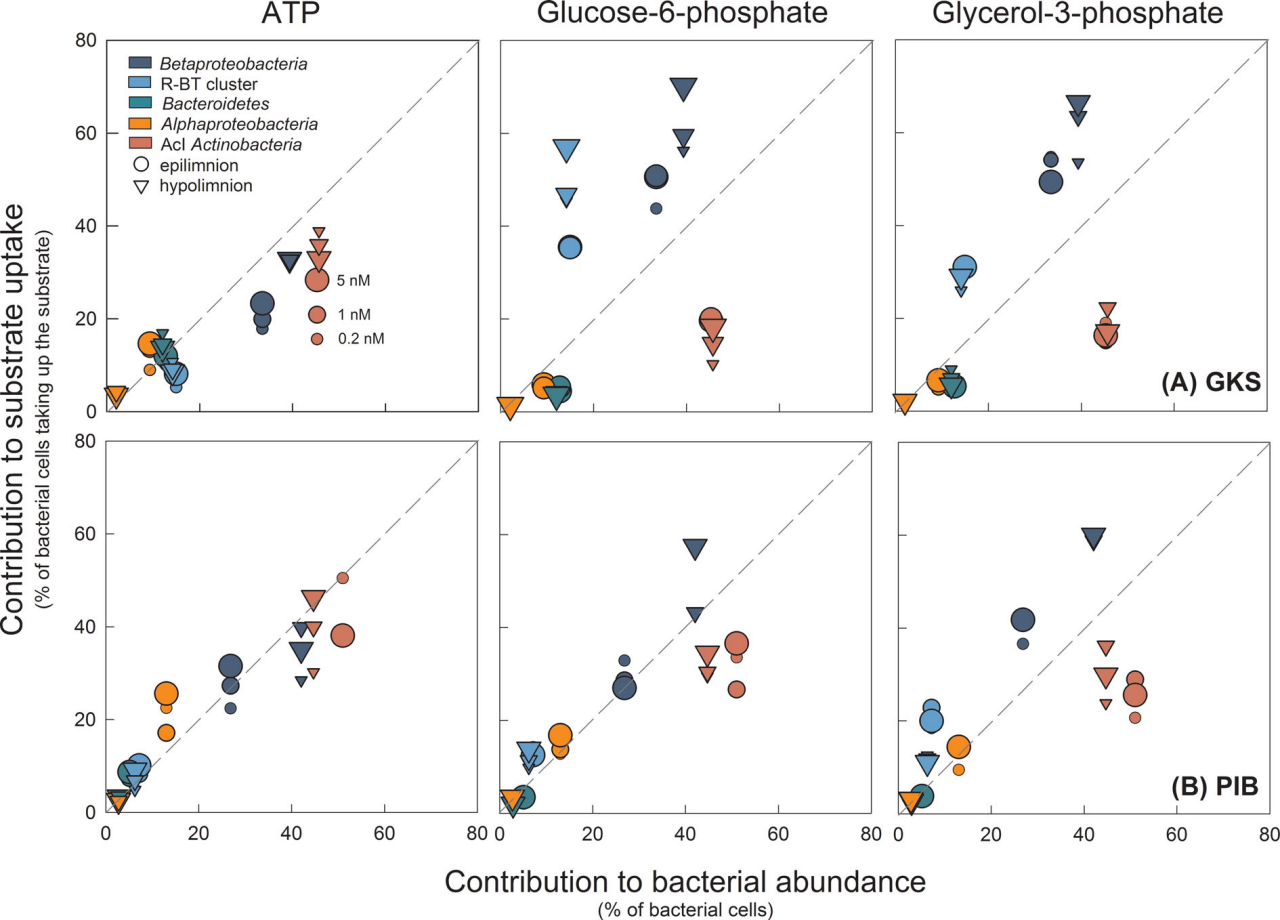
The contribution of *Betaproteobacteri*a (dark blue) and its R-BT cluster (light blue), *Bacteroidetes* (green), *Alphaproteobacteria* (orange), and AcI *Actinobacteria* (red) to ATP, glucose-6-phosphate and glycerol-3-phosphate uptake plotted against their contribution to abundance in GKS (**A**) and PIB (**B**). The 1:1 line indicates that the contribution of a bacterial group to substrate uptake equals its contribution to bacterial abundance. Circle and triangle indicate the epilimnion and hypolimnion, respectively. Different substrate concentrations are given by symbol size: small, 0.2 nM; medium, 1 nM; large, 5 nM. Values are the mean of triplicate samples.

The contribution of a bacterial group to the uptake of ATP, Glu6P and Gly3P versus its contribution to bacterial abundance differed depending on the substrate and lake considered (Fig. 3A and B). *Alphaproteobacteria* usually contributed to the uptake of the three DOP compounds proportionally to their *in situ* abundance, though they were slightly over-represented in the uptake of ATP in the epilimnion of both lakes. *Betaproteobacteria* and its R-BT cluster were significantly over-represented in the uptake of Glu6P and Gly3P as compared with their *in situ* abundance in GKS (ANOVA, *P* < 0.01), whereas their contribution to ATP incorporation was proportional to their abundance. Similarly in PIB, they were close to the 1:1 line for ATP and Glu6P uptake, but were over-represented for Gly3P (ANOVA, *P* < 0.05). *Bacteroidetes* contributed to substrate incorporation in relation to their relative abundance, except in GKS where they were slightly under-represented in the uptake of Glu6P and Gly3P. AcI *Actinobacteria* were poorly represented in the uptake of Glu6P and Gly3P. This trend was significantly more pronounced in GKS than in PIB (*t*-test, *P* < 0.001). Their contribution to ATP uptake was rather close to the 1:1 line in both lakes (Fig. 3).

## DISCUSSION

### Dissolved organic phosphorus compounds readily utilizable by lake bacteria

The dissolved organic phosphorus (DOP) pool comprises a variety of different chemical compounds. The bioavailable DOP fraction is constituted by nucleotides, phosphoesters (Berman 1988; Björkman and Karl 1994) and phosphonates (Schowanek and Verstraete 1990). Among DOP, nucleotides such as ATP seem to be the most labile molecules as suggested by their rapid turnover times of hours (Bentzen, Taylor and Millard 1992; Løvdal, Tanaka and Thingstad 2007). However, P-limited bacteria may rely on multiple DOP compounds to satisfy their P demand. In fact, in most cases we observed similar proportions of bacterial cells labeled positive for the uptake of the three DOP substrates considered: ATP, Glu6P and Gly3P. However, the specific bulk uptake rates of those DOP compounds differed, as well as their kinetic patterns (Fig. 2A and B). For instance, the uptake rates for Glu6P and Gly3P increased with increasing concentrations without reaching saturation at 5 nM, whereas for ATP they remained rather constant or fluctuated without clear pattern. This suggests that even higher concentrations of Glu6P and Gly3P could be taken up by heterotrophic bacterial communities and that these compounds are readily utilized by heterotrophic bacteria, probably due to the P-deficient conditions in both lakes. However, it has to be noted that in this study P uptake was not tracked directly because the DOP compounds we used were ^3^H-labeled. Nonetheless, considering previous studies on ATP, Glu6P and Gly3P uptake mechanisms, we could infer the most likely P uptake mechanisms working in both lakes. For instance, the [^3^H]ATP uptake rates we measured were similar to rates found in other studies (Cotner and Wetzel 1992; Alonso-Sáez and Gasol 2007; Alonso-Sáez, Sánchez and Gasol 2012), but were one order of magnitude lower than [^33^P]ATP rates measured in the same lakes previously (Rofner, Sommaruga and Pérez 2016, in press). Significantly higher bulk uptake rates of [^33^P]ATP compared with [^3^H]ATP were also observed by Duhamel, Björkman and Karl (2012). This large discrepancy in ATP uptake rates can be explained by the fact that three orthophosphate moieties need to be hydrolyzed before the [^3^H]adenine moiety can be taken up. Thus, the adenine moiety is taken up much slowly than the orthophosphate residues (Heath and Hansen 2004), and thus the ATP uptake rates we report here might underestimate the uptake of orthophosphate. Considering this fact, we expected ATP uptake rates to be lower than Glu6P and Gly3P uptake rates. However, the bulk uptake rates were in a similar range for all three substrates, suggesting that ATP is being utilized rapidly in both lakes.

Previous studies on the mechanisms involved in Glu6P uptake in *E*. *coli* showed that, under P-sufficient conditions, this substrate is taken up directly via the UhpT system (antiporter) by exchanging P_i_ or organophosphates against Glu6P (van Veen 1997). This mechanism, however, would be very inefficient under P-limiting conditions, and thus Hoffer, van Uden and Tommassen (2001) suggested that under P limitation, extracellular alkaline phosphatases would hydrolyze Glu6P and that the orthophosphate moiety would be incorporated separately from the C moiety. Indeed, the study of Hernandez, Hwang and Heath (1996) in a mesotrophic lake showed that the uptake of the orthophosphate moiety is much faster than the uptake of the C moiety, by comparing [^32^P]Glu6P with [^14^C]Glu6P uptake rates. Thus, the [^3^H]Glu6P bulk uptake rates we measured might underestimate the incorporation of organic phosphorous from Glu6P. However, the fact that increasing substrate concentrations resulted in higher Glu6P uptake rates suggests that in the study lakes bacteria profited from both the glucose and the orthophosphate moiety.

Among the three DOP compounds studied, Gly3P yielded the highest bulk uptake rates, particularly in the oligotrophic alpine lake GKS (Fig. 2A and B). Under P-limiting conditions, Gly3P is incorporated as a whole by the ugp system (Schweizer, Argast and Boos 1982; Brzoska *et al.*^1994^; Luo *et al.*^2009^; Vila-Costa *et al.*^2012^), which means that the C and orthophosphate moiety are taken up at the same time. The high uptake rates found in both lakes are likely due to the fact that Gly3P does not need to be hydrolyzed prior to uptake. Furthermore, the ugp transport system, connected to the Pho regulon, is controlled by intracellular P_i_ levels (Brzoska *et al.*^1994^) and the higher Gly3P uptake rates in GKS when compared with PIB may be due to the particular low P_i_ concentrations found in this lake (Rofner, Sommaruga and Pérez 2016).

### Dissolved organic phosphorus uptake by individual bacterial groups

In P-limited aquatic ecosystems, the activity and dynamics of bacteria are undoubtedly affected by their inherent and adaptive ability to acquire P compounds. Several ‘omic’-based and single-cell studies have revealed that distinct gene expression profiles and substrate uptake patterns prevail among different bacterial groups/subgroups/clusters (Alonso-Sáez and Gasol 2007; Luo *et al.*^2009^; Longnecker, Lomas and Van Mooy 2010; Sebastián *et al.*^2012^; Vila-Costa *et al.*^2012^), but knowledge on what bacterial groups dominate the uptake of different DOP compounds is still missing.

In our study, we identified DOP-specific uptake patterns for the main bacterial groups/subgroups inhabiting two mountain lakes (Fig. 1) and found that the most abundant taxa, the AcI *Actinobacteria* and the *Betaproteobacteria*, exhibited strikingly different DOP uptake characteristics. Whereas *Betaproteobacteria* and particularly its R-BT cluster (genus *Limnohabitans*), were the most active in acquiring Glu6P and Gly3P, AcI *Actinobacteria* were rather under-represented in the uptake of all DOP compounds considered (Fig. 3). This does not necessarily imply that the AcI *Actinobacteria* are less competitive in DOP acquisition than the R-BT cluster, but indicates that these subgroups follow distinct strategies to cope with P-limiting conditions. Freshwater bacteria exhibit striking flexibility in their P content (Godwin and Cotner 2015) and in their growth potential (Šimek *et al.*^2006^). The growth rate hypothesis (Sterner 1995) postulates that slow growing cells will have a lower P content than rapid growing ones. The weak representation of AcI *Actinobacteria* in DOP uptake could be due to their moderate growth rates (Šimek *et al.*^2006^) and, thus, low P requirements. An alternative explanation is that they replace membrane phospholipids with non-phosphorus lipids (e.g. glycolipids) to reduce their P demand as shown for the marine alphaproteobacterial clade SAR11 (Sebastián *et al.*^2012^; Carini *et al.*^2015^). Although some lipase and glycosyltransferase genes involved in cell wall biogenesis of AcI representatives are present (Ghylin *et al.*^2014^), further studies are needed to elucidate whether AcI *Actinobacteria* are capable of membrane remodeling. However, their weak representation in Glu6P and Gly3P incorporation might also be linked to a limited number of gene sites coding for DOP uptake, as AcI *Actinobacteria* are known to have small and streamlined genomes (Ghylin *et al.*^2014^). This is supported by information obtained from a P-limited alpine lake in the Pyrenees (Vila-Costa *et al.*^2012^), where *Actinobacteria* contributed little to the transcript pool of P-related functional genes (ugp transport system, alkaline phosphatases, phosphonate uptake). However, we cannot exclude the possibility that AcI *Actinobacteria* obtain P from other DOP compounds not considered here.

Unlike AcI *Actinobacteria*, *Betaproteobacteria* were significantly over-represented in the uptake of Glu6P and Gly3P in the oligotrophic lake (GKS) and occasionally in the mesotrophic PIB (Fig. 3). In both lakes, we generally observed that the proportions of betaproteobacterial cells taking up DOP substrates increased with increasing substrate concentrations. This pattern agrees with the fact that freshwater *Betaproteobacteria* are rapidly enriched in response to an increase in the organic and inorganic P concentration (Burkert *et al.*^2003^; Šimek *et al.*^2005^; Hornák *et al.*^2006^; Pérez and Sommaruga 2006; Posch *et al.*^2007^). Depending on the aquatic system considered, different betaproteobacterial clades were responsible for the enrichment. The fast-growing R-BT cluster (beta I lineage) (Šimek *et al.*^2006^), which is ubiquitous in neutral to alkaline lakes (Šimek *et al.*^2010^), became very abundant in the meso-eutrophic Římov Reservoir (Hornák *et al.*^2006^) and in the alpine lake GKS (Pérez and Sommaruga 2006), whereas the *Polynucleobacter necessarius* clade (beta II lineage) was rapidly enriched in humic lakes (Burkert *et al.*^2003^; Hahn, Pöckl and Wu 2005). In GKS, the R-BT cluster usually dominates within the *Betaproteobacteria* (Pérez and Sommaruga 2011), whereas in PIB, lineages such as beta II and IV are also present (Salcher *et al.*^2008^), but they only reach a high representation in the anoxic hypolimnion.

In both lakes, we found that the proportions of R-BT cells incorporating DOP compounds followed the uptake patterns found at the group level (*Betaproteobacteria*). As much as 95% and 77% of R-BT cells were labeled positive for Glu6P and Gly3P uptake, respectively (Fig. 2C and D). This shows that the R-BT cluster is particularly important in the cycling of phosphorylated sugars and glycerol compounds. Indeed, when we screened the available information on putative genes involved in DOP uptake for *Limnohabitans* from the Římov Reservoir (accession number NCBI: SAMN02470021, SAMN02470022), we found several genes related to glycerol-3-phosphate uptake (*ugpQ, ugpC*) and phosphoester hydrolysis (phosphatases), as well as to P_i_ starvation (*phoH, phoR, phoU*) and P_i_ uptake (*pstA, pstC, pstS*). Additionally, both genomes showed high frequency of genes involved in phosphonate metabolism, which points to the R-BT cluster playing an important role in the utilization and cycling not only of phosphoesters but also of phosphonates in freshwaters.

*Alphaproteobacteria* and *Bacteroidetes* are usually not the most abundant bacterial groups in oligotrophic freshwaters (Pérez, Hörtnagl and Sommaruga 2010; Salcher, Pernthaler and Posch 2010). However, they prevail at specific periods during the year (Cottrell and Kirchman 2000; Eiler *et al.*^2003^; Pinhassi and Berman 2003). A metatranscriptomic study found that *Alphaproteobacteria* and *Bacteriodetes* from an alpine lake express several transcripts related to DOP uptake (ATP, Glu3P, phosphonate uptake) (Vila-Costa *et al.*^2012^) suggesting that they might contribute substantially to DOP cycling.

*Bacteroidetes*, which are often under-represented in the uptake of low-molecular-weight compounds (Cottrell and Kirchman 2000; Salcher, Posch and Pernthaler 2013), have, nevertheless, been found to harbor high proportions of cells positive for the uptake of P-containing substrates (Alonso-Sáez and Gasol 2007; Longnecker, Lomas and Van Mooy 2010; Sebastián *et al.*^2012^; Pérez, Rofner and Sommaruga 2015). Similarly, previous studies revealed that *Alphaproteobacteria* contribute to ATP uptake proportionally to their abundance (Alonso-Sáez and Gasol 2007; Sebastián *et al.*^2012^; Pérez, Rofner and Sommaruga 2015; Rofner, Sommaruga and Pérez 2016) and also that they account for the majority of ugp-transporter genes responsible for Gly3P uptake in marine waters (Luo *et al.*^2009^). Our study confirms that *Alphaproteobacteria* and *Bacteroidetes* utilize beside ATP, Glu6P and Gly3P, and shows that in P-limited systems, they contribute to DOP uptake proportionally to their *in situ* abundance (Fig. 3).

## CONCLUSIONS

Here, we have characterized the strategies of P-limited freshwater bacteria to acquire DOP compounds. We showed that beside ATP, Gly3P and Glu6P are readily taken up by freshwater bacteria, which proves that they can efficiently use DOP compounds at a wide range of naturally occurring substrate concentrations. Additionally, we found that the most abundant bacterial groups, namely, AcI *Actinobacteria* and *Betaproteobacteria*, exhibited strikingly different DOP uptake patterns. The R-BT cluster of *Betaproteobacteria* was the most active in acquiring Glu6P and Gly3P indicating that they dominate the uptake of low-molecular-weight DOP compounds. By contrast, AcI *Actinobacteria* were less involved in DOP acquisition, because either they lack efficient DOP uptake systems or they exhibit low P requirements.
